# Economic Evaluation of Cement Grouted Bituminous Mixes for Airport Pavements

**DOI:** 10.3390/ma14237230

**Published:** 2021-11-26

**Authors:** Paola Di Mascio, Giuseppe Loprencipe, Laura Moretti

**Affiliations:** Department of Civil, Constructional and Environmental Engineering, Sapienza University of Rome, 00184 Rome, Italy; paola.dimascio@uniroma1.it (P.D.M.); giuseppe.loprencipe@uniroma1.it (G.L.)

**Keywords:** open-graded asphalt, cement grouted open graded, CGBM, airport pavement, helipad, heliport pavement

## Abstract

The Cement Grouted Bituminous Mix (CGBM) is an innovative material that could be used to build airport pavements subjected to heavy concentrated loads or fuel and solvent leaks. CGBM is composed of a porous asphalt clogged with an expansive cement mixture, which fills the asphalt voids. This paper focuses on two airport pavements (i.e., a taxiway and a helipad one) to be paved in an Italian airport. For each surface, the construction and maintenance costs of a CGBM pavement and a traditional flexible pavement have been compared. The pavements should bear different traffic loads, while the weather, subgrade, and materials are the same: the fatigue and rutting verification gives structures whose cost analysis leads to different results. The CGBM solution for the taxiway has a cost comparable to that of the equivalent traditional flexible pavement (i.e., 73.87 €/m^2^ vs. 73.20 €/m^2^ during the service life). On the other hand, the overall discounted cost of the helipad surface paved with CGBM is higher than that obtained for the traditional pavement (i.e., 82.4 €/m^2^ vs. 67.5 €/m^2^). Therefore, the study demonstrates that the economic opportunity of CGBM solutions strongly depends on traffic loads.

## 1. Introduction

In the last decades, research in the field of road and airport pavements is pointing to materials that guarantee operating safety (e.g., adhesion and surface regularity), long service life (e.g., durability), low maintenance costs (e.g., less failures, fewer ruts and cracks), and low environmental impacts (e.g., use of secondary raw materials and adoption of low emitter processes) [1,2,3]. The open-graded asphalt filled with a special cement grout is part of a line of research aimed to design pavements with innovative materials [4]. Cement Grouted Bituminous Mix (CGBM) has been used to solve durability problems in areas subject to heavy concentrated loads or fuel and solvent leaks [5]. In the literature, port storage areas, surfaces where aircraft move, and parks for heavy vehicles, both for construction and maintenance works, demonstrated the opportunity to use CGBM in the upper layer of the pavement [6]. Recently, important applications in the airport field occurred [7,8,9]. Airport pavements substantially differ from road ones due to traffic loads (both mass and frequency of repetitions) and environmental aggressions (e.g., chemical attack and temperatures) [10]. Indeed, on road pavements the heaviest axle load is of the order of 100 kN (i.e., a coupling of two 50 kN wheels), while in airport pavements landing gears can apply loads in excess of 900 kN (e.g., a B747-400) with high tire inflation pressure (e.g., 1.4 MPa for the tires of a B747 compared to 0.6–0.7 MPa for a heavy road vehicle). On the other hand, the number of repetitions during the service life is generally much lower than on the road (the busiest Italian airport has approximately 300,000 movements/year on three runways, while a road with average traffic can count 1,000,000 passes/year). Moreover, dynamic forces cannot be overlooked in the aeronautics context: in airports, the speed depends on the type of infrastructure (runway, taxiway, and apron) and on the section of the runway, while along a road network it does not change significantly [11]. On a runway, aircraft speed reaches up to 250 km/h, while the speed is low (10–30 km/h) and constant on taxiways; usually, the maximum allowable speed on highways is not more than 130 km/h for cars and less than 90 km/h for trucks. Therefore, airport pavements where aircraft move are thicker than road pavements to withstand both greater static and dynamic stresses under traffic. Airport pavements are usually composed of asphalt for the central part of runways and traffic routes, and concrete slabs where there may be aircraft fuel leaks (which, as it is known, contains solvent substances for bitumen), or where there may be harmful effects due to the jet of the engines (e.g., runway thresholds and aprons). On the other hand, slow movements and stationing on aprons could result in rutting and force to structural solutions alternative to flexible ones [12]. However, airport pavements include heliport surfaces whose traffic loads are more similar to road vehicles than aircraft. Therefore, alternative solutions are under study to evaluate their cost-effectiveness.

**Figure 1:**
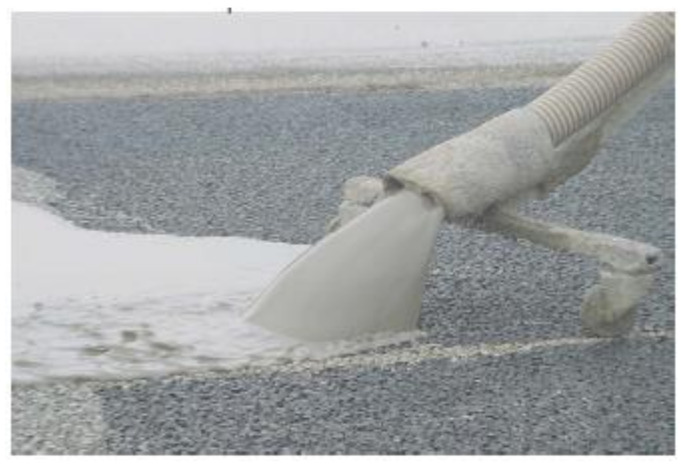
Cement mortar spreading.

A cement grouted open graded asphalt pavement is composed of a porous asphalt, which constitutes the surface, and of an expansive cement mixture, which clogs the voids [9]. After the asphalt laying, the compaction is carried out using 8–10 t static metal rollers to reach the maximum degree of contact between the stone elements while retaining the network of intergranular voids reached at the end of the paving with paver. The compaction process requires a double passage of the roller within a period of 10 min since the laying, so that the temperature of the material does not decrease by more than 10 °C compared to the spreading one. Before laying, a tack coat of bituminous emulsion is applied, at the rate of 1–2 kg/m^2^, to have an impermeable contact surface and to avoid infiltration of the clogging mortar into the bottom layers. Then, voids are clogged with an expansive cement mortar to counteract shrinkage cracks. The cement mortar is spread [13] (Figure 1) under specific conditions: the injection starts at least 12 h after laying, when the porous asphalt has reached room temperature (not more than 35–40 °C). Indeed, the temperature of the bituminous layer strongly influences the viscosity of the mortar and consequently its workability, and water evaporation.

The obtained continuous surface [14] gives an interesting performance in terms of stiffness and static punching loads, and very high load-bearing capacity, resistance to chemical aggression, petrol and intense heat [15]. This pavement type ensures the durability of areas where heavy and slow loads are applied, or where fuel and solvent leaks: from port storage areas, to parking lots for heavy loads, and recently there have been important applications in the airport field (e.g., taxiways and helipads). In the airport network, open grade pavements can be used for taxiways, aprons, push-back areas, holding bays, de-icing and refueling areas, and runway thresholds [7]. On the other hand, their use for runways is not recommended because it is difficult to reach the required adhesion [16]. Porous asphalt has generally 25–30% voids and 4–5% by volume of bitumen. The high percentage of voids is due to a discontinuous grading curve of crushed aggregates, and affects density values ranging between 1.8–1.9 g/cm^3^. The binder used is natural or modified bitumen (modified bitumen is obtained from a natural one whose chemical structure and properties are modified by adding suitable materials, e.g., polymers, additive [17]).

In this paper, two CGBM pavements have been designed to pave taxiway and helipad surfaces in an Italian airport, and are compared with equivalent asphalt pavement with regard to their construction and maintenance costs. The surfaces differ for traffic loads, which significantly affect the obtained solution and its economic performance during the service life. It leads to two different solutions, although the weather, subgrade, and materials input data coincide. The obtained results highlight that the CGBM solution is competitive for the examined taxiway, while it is more expensive in regard to the helipad surface.

## 2. Materials and Methods

Table 1 lists the physical and mechanical characteristics of the bitumen used to produce the studied porous asphalt layer.

The grading curve of aggregates should ensure in the asphalt matrix a network of communicating intergranular voids: in this study, the selected aggregates have voids in the mineral aggregate (VMA) equal to 28%. VMA is the volume available to accommodate bitumen and then the injected mortar. The maximum diameter of aggregates has to be studied in order to apply a 4–8 cm-thick open grade; mechanical and physical requirements of aggregates are in Table 2.

The cement mortar used in a CGBM pavement is a mixture of hydraulic binders and additives, premixed in powder, to be mixed with water, without chlorides, hyperfluid, with high penetration and void filling capacity. Table 3 lists the mix design of the cement mortar used to fill the voids of the porous asphalt layer under study.

Table 4 lists the physical and mechanical properties of a CGBM layer: the mutual collaboration between stone aggregates and cement mortar ensures a continuous answer to the stresses.

In both areas to be paved, the subgrade loading capacity is good (i.e., California Bearing Ratio (CBR) equal to 20%). The input data about traffic are listed in Table 5 for the taxiway, and in Table 6 for the helipad; yearly movements refer to the construction year, and an annual increase rate equal to 5% has been assumed.

A traditional asphalt pavement has been designed for the taxiway according to the Federal Aviation Administration (FAA) Airport Pavement Design and Evaluation [26], and then it has been verified according to the elastic multilayer theory [27] with regard to the fatigue distress for bound layers (Equation (1)) and rutting distress for granular materials (Equation (2)) [28]:

(1) $$N_{c}=f_{1}\cdot \varepsilon^{-f_{2}}\cdot {\left|E^{*}\right|}^{-f_{3}}$$

(2) $$N_{s}=f_{4}\cdot \varepsilon_{c}{}^{-f_{5}}$$

where *N_c_* is the number of repetitions that causes the fatigue rupture, *ε* is the maximum horizontal deformation at the bottom of the asphalt layers, E* is the asphalt elastic modulus, *N_s_* is the number of repetitions that causes permanent plastic deformations of the granular layers and the subgrade, *ε_c_* is the vertical deformation at the bottom of the unbound layers or the subbase layer, and *f*_1_, *f*_2_, *f*_3_, *f*_4_, *f*_5_ are experimental coefficients (Table 7) provided by the Shell and Asphalt Institute [28].

Both verifications have been carried out according to the Miner’s law (Equation (3) [29]):

(3) $$\sum_{i}\frac{n_{i}}{N_{i}}\leq 1$$

where *n_i_* is the number of expected repetitions of the *i*-th load during the service life and *N_i_* is the number of repetitions of the *i*-th load that cause the failure.

Elastic moduli of the pavement materials were obtained from the software FAARFIELD 1.42 (Federal Aviation Administration Rigid and Flexible Iterative Elastic Layered Design) (Federal Aviation Administration, Washington, DC, USA, 2017) [26].

Previous studies carried out by the authors gave information about construction and maintenance costs for traditional asphalt and CBGM pavement; materials, machines and labors are considered in the analysis according to the unit price values adopted in this study. The over-time cost analysis was carried out considering maintenance activities and assuming an interest rate of 3% and an inflation rate of 1.5% to have a Discounted Cash Flow Analysis. The analysis did not take into account ordinary maintenance costs (e.g., degumming) because they are the same for both pavements, and therefore do not affect the economic evaluation of the alternatives. Maintenance works, on the other hand, were scheduled and assessed based on the past experience of airport infrastructure managers. Calculation of the discounted M&R cost in the specific year during the analyzed period complies with the economic model proposed in the Technical Manual No. 5–623 Pavement Maintenance Management [30]. Therefore, for each pavement the discounted costs refer to the overall costs at the construction year obtained deducting the costs during the service life for interest determined by the time and by risk [31].

## 3. Results

### 3.1. Taxiway Pavement

Table 8 lists the output for airport pavements obtained using the software FAARFIELD 1.42 [30].

Shell curves give the most severe results in terms of pavement service life (Table 9).

**Figure 2:**
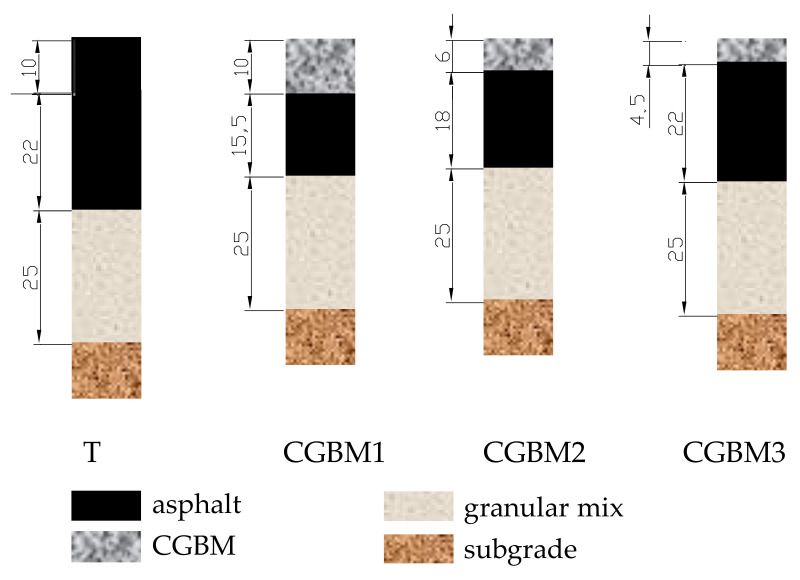
Compared taxiway pavements (units in cm).

In order to have CGBM service life equal to that obtained for the traditional pavement, an iterative analysis was carried out considering the mechanical performances (i.e., results from fatigue and rutting curves), economic issues (i.e., construction costs), and operational needs (i.e., minimum CGBM thickness equal to 6 cm). Table 10 summarizes the compared solutions for the taxiway pavement (Figure 2): they differ for upper layers thickness.

**Figure 3:**
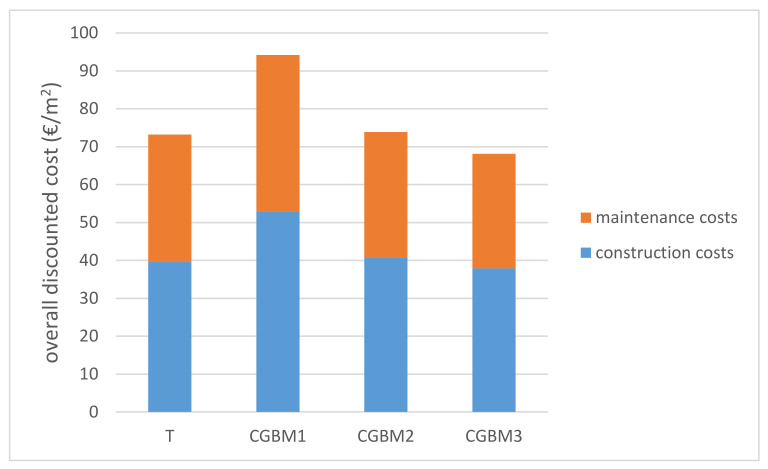
Overall discounted costs.

Construction costs highlight that, for the same service life, CGBM pavements are more expensive than traditional ones. Maintenance costs were obtained from airport pavement management systems (APMS) developed and implemented in two Italian airports, herein not disclosed due to privacy reasons. Therefore, the maintenance costs take into account all maintenance and rehabilitation costs to counter distresses for asphalt and CGBM pavement. Figure 3 shows the results of the overall discounted pavement costs.

The analysis of both construction and maintenance costs demonstrated the cost-effectiveness of CGBM2 and CGBM3: their overall discounted costs are comparable to that obtained for T (i.e., 73.87 €/m^2^ and 68.12 €/m^2^ vs. 73.20 €/m^2^). However, the cheapest option (i.e., CGBM3) is composed of 4.5 cm of open graded asphalt, and it is not feasible due to low thickness of the upper layer. Therefore, CGBM2 is the best solution alternative to the traditional asphalt pavement because it has the lowest present value (i.e., it requires the lowest investment by the pavement manager).

### 3.2. Helipad Pavement

A different approach was implemented to design the helipad pavements: the CGBM pavement was designed with a 6 cm-thick open graded layer. This thickness was chosen because of the lower traffic loads (Table 6) and the technical constraints (i.e., non-feasibility of less than 6 cm-thick layers). Therefore, the study started from a helipad pavement composed of:

- 6 cm-thick CGBM;
- 25 cm-thick cement bound base;
- 30 cm-thick granular subbase;
- subgrade.

Its verification process was carried out according to the elastic multilayer theory [27] with the software BISAR 3.0 (1998, Bitumen Business Group, Moscow, Russia) [32]. Mechanical characteristics of layers are listed in Table 11.

Radial and vertical stresses have been taken into account for the bound layers of the CGBM pavement: Table 12 lists the compressive (minus sign) and tensile (plus sign) strength of the bound layers.

**Figure 4:**
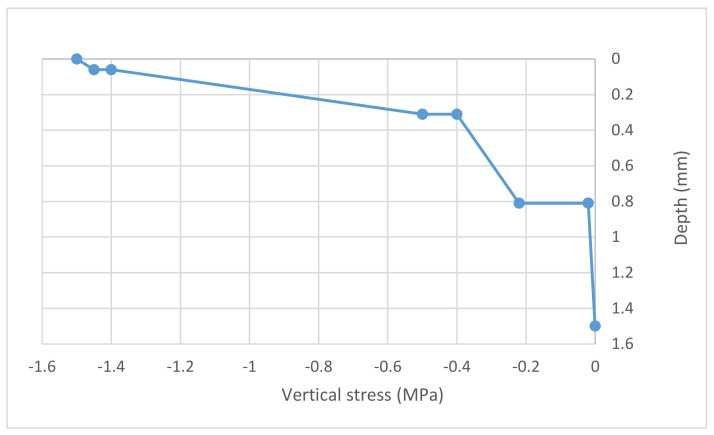
Maximum vertical stress under load—CGBM pavement.

**Figure 5:**
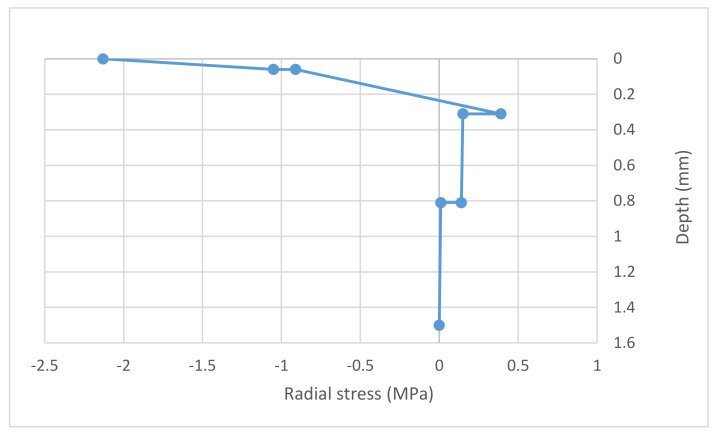
Maximum radial stress under load—CGBM pavement.

Figure 4 and Figure 5 show the maximum vertical and radial stresses, respectively, obtained from verification of the CGBM pavement.

Both the maximum vertical and the maximum radial calculated stresses do not exceed the mechanical strength of the bound materials in Table 12: the CGBM pavement is verified. This pavement has been verified in regard to fatigue and rutting of unbound and bound layers (Equations (1) and (2)) [26]. According to Shell curves [28], its allowable number of repetitions is 377,751. That value has been considered to design the equivalent asphalt pavement that it is composed of:

- 10 cm-thick asphalt;
- 28 cm-thick cement bound base;
- 30 cm-thick crushed aggregate subbase;
- subgrade.

Table 13 shows the economic comparison between the verified heliport pavements.

The overall discounted costs calculated for traditional and CGBM helipad pavements demonstrated the verified solution with wearing open grade asphalt clogged with cement mortar is more expensive than the equivalent solution with wearing asphalt layer. This result needs to be interpreted: it is not possible to affirm that a CGBM pavement is not a good solution, because a pavement manager should examine several challenges. In some particular situations, it might be convenient to minimize maintenance interventions in order not to interfere with the operation of the heliport. The clogging mortar is prepared in-situ by means of a mobile plant; given a service life period, CGBM pavements are thinner than traditional asphalt pavements: it implies reduction of raw or recycled materials; its skeleton avoids rutting induced by severe thermal conditions in traditional asphalt pavements; on the other hand, its continuous surface avoids joint sealing and maintenance needed for concrete slab pavements.

This study has been obtained in regard to Italian input data (e.g., materials, weather, unit costs): the economic conclusions may differ based on national circumstances because it is affected by both unit prices and weather conditions. However, the proposed approach could be repeated under different conditions to compare pavement solutions, not just for helipads.

## 4. Conclusions

Cement Grouted Bituminous Mix (CGBM) consists of an open grade asphalt clogged with expansive cement mortar that clogs the voids of the asphalt. In the last decades this solution has been used in port and airport areas subject to heavy loads or where fuel and solvent leaks can occur: port storage areas, parking lots for heavy loads, airport taxiways and apron, and helipads.

This paper deals with two CGBM pavements designed to pave a taxiway and a helipad in an Italian airport. The taxiway pavement is composed of 6 cm thick CGBM, 18 cm asphalt base, and 25 cm granular subbase. The traffic includes medium size aircraft whose loads and yearly movements are listed in Table 5. The helipad pavement is composed of a 6 cm thick CGBM layer, a 25 cm thick cement bound base, and 30 cm thick cement bound subbase over the subgrade. The traffic is composed of different types of helicopters with loads and yearly movements listed in Table 6. The subgrade CBR is equal to 20% in both cases.

The stresses and strains of the above structures were calculated according to the elastic multilayer theory, and the pavements were verified with the Miner’s law, calculating the allowable number of repetitions before fatigue rupture in bound layers and rutting in unbound layers. The overall discounted construction and maintenance costs were calculated for the two CGBM pavements, and each one has been compared with the equivalent traditional flexible pavement composed of a 10 cm-thick wearing course. Under the wearing course, the taxiway pavement is composed of 22 cm thick asphalt concrete base and 25 cm thick granular subbase, while in the helipad pavement 28 cm thick cement bound base and 30 cm thick cement bound subbase are laid. The obtained results highlight that the costs of the CGBM solution are comparable to the traditional pavement for taxiway, while the CGBM pavement is more expensive in regard to the helipad surface.

However, CGBM has several advantages during its life: the clogging mortar is mixed in-situ by means of a mobile plant; because the CGBM pavements are thinner than traditional ones, a reduction in raw or recycled materials is possible; their skeleton avoids rutting induced by severe thermal conditions in traditional asphalt pavements; their continuous surface avoid joint sealing and maintenance needed for concrete pavements.

In conclusion, CGBM is an alternative to both asphalt and concrete, combining the characteristics of both. It ensures longer durability, lower temperature susceptibility, and more resistance to fuel spillage. It has a high load-bearing capacity like rigid pavements, but has the costs and speed of laying more similar to flexible ones, and it requires less maintenance than the flexible ones.

## Figures and Tables

**Table 1:** Characteristics and quantity of bitumen.

Property	Unit of Measure	Natural Bitumen		Modified Bitumen	
penetration grade at 25 °C [18]	dmm	50–70	80–100	50–70	50–70
Softening point [19]	°C	46–56	40–44	>65	>60
Fraass breaking point [20]	°C	<−10	<−8	<−15	<−12
Elastic recovery at 25 °C [21]	%	>80	>80	>75	>50
bitumen content by volume	%	3–4			

**Table 2:** Characteristics of aggregates.

Property	Unit of Measure	Value
Los Angeles coefficient [22]	%	<22–25
Fractured particles in Coarse Aggregate [23]	%	100
Flakiness index [24]	%	<20
Bulk density [25]	g/cm^3^	>2.6

**Table 3:** Characteristics of a CGBM.

Material	Quantity [kg]
Cement	450
Water	180
Sand	1350
Expansive additive	2.25

**Table 4:** Characteristics of a CGBM.

Parameter	Unit of Measure	Value
Compressive strength at 1 day	MPa	4–7
Compressive strength at 7 days	MPa	7–10
Compressive strength at 28 days	MPa	8–12
Elastic modulus	MPa	8000–12,000
Coefficient of linear thermal expansion	1/°C	12.5 × 10^−6^
Density	g/cm^3^	2.32
Indirect tensile strength	MPa	3.28 (T = 10°)
		1.99 (T = 25°)
		1.15 (T = 40°)

**Table 5:** Taxiway traffic.

Aircraft	Maximum Take-Off Mass (kg)	Wheel Load (kg)	Yearly Movements (-)
CRJ-900	37,421	8888	170
A320	80,000	19,000	1331
A319	77,000	18,288	1331
B737-800	74,000	17,575	16,338
B737-700	67,000	15,913	147
B737-500	59,000	14,013	408
B737-400	62,500	14,844	498
B737-300	59,500	14,131	2753

**Table 6:** Helipad traffic.

Helicopter Model	Maximum Take-Off Mass (kg)	Yearly Movements (-)
HH 212	5080	189
HH 101A	15,600	467
AW 139	6800	328
TH 500B	1361	123
AB 412	5298	437
AC 145 Cw	3585	325

**Table 7:** Fatigue and rutting coefficients.

Source	*f*_1_	*f*_2_	*f*_3_	*f*_4_	*f*_5_
Shell	0.0685	5.671	2.363	6.155 × 10^−7^	3.571
Asphalt Institute	0.0796	3.291	0.854	1.365 × 10^−9^	4.477

**Table 8:** Taxiway pavement.

Layer Material	Thickness (mm)	Modulus (MPa)	Poisson Coefficient (-)
Traditional asphalt (or open graded)	100	1378 (8000)	0.20
Asphalt	220	2757	0.20
Granular mix	250	1723	0.35
Subgrade	CBR = 20%	206	0.35

**Table 9:** Expected service life.

Pavement Type	Number of Repetitions	Service Life Duration (Years)
Traditional asphalt	710,060	33.4
CGBM	1,139,739	53.7

**Table 10:** Construction costs of the compared taxiway pavements.

Pavement Type	ID	Upper Layers Thickness (cm)	Construction Costs (€/m^2^)
Traditional asphalt	T	10 + 22 = 32	39.68
CGBM	CGBM1	10 + 15.5 = 25.5	52.87
	CGBM2	6 + 18 = 24	40.78
	CGBM3	4.5 + 22 = 26.5	37.91

**Table 11:** Helipad pavement.

Layer Material	Thickness (mm)	Modulus (MPa)	Poisson Coefficient (-)
CGBM	60	7000	0.20
Cement bound mix	250	4000	0.30
Granular mix	300	2000	0.30
Subgrade	∞	40	0.35

**Table 12:** Values of compressive and tensile strength of the bound layers.

Layer Material	Horizontal Radial Stress Limit (MPa)	Vertical Stress Limit (MPa)
CGBM	−5.1 +1.7	−12.5
Lean concrete	−1.5 +0.5	−4.0

**Table 13:** Construction and maintenance costs of the compared helipad pavements.

Pavement Type	Construction Costs (€/m^2^)	Overall Discounted Costs (€/m^2^)
Traditional asphalt	34.7	67.5
CGBM	51.4	82.4

## Data Availability

Data is contained within the article.
